# The tankyrase inhibitor G007-LK inhibits small intestine LGR5^+^ stem cell proliferation without altering tissue morphology

**DOI:** 10.1186/s40659-017-0151-6

**Published:** 2018-01-09

**Authors:** Jens Henrik Norum, Ellen Skarpen, Andreas Brech, Raoul Kuiper, Jo Waaler, Stefan Krauss, Therese Sørlie

**Affiliations:** 1 Department of Cancer Genetics and SFI CAST, Institute for Cancer Research, Oslo University Hospital, The Norwegian Radium Hospital, 0310 Oslo, Norway; 2 Department of Molecular Cell Biology, Institute for Cancer Research, Oslo University Hospital, The Norwegian Radium Hospital, 0310 Oslo, Norway; 3 Department of Core Facilities, Oslo University Hospital, The Norwegian Radium Hospital, 0310 Oslo, Norway; 4 Department of Biosciences, University of Oslo, 0310 Oslo, Norway; 5 Centre for Cancer Biomedicine, 0310 Oslo, Norway; 6 Department of Laboratory Medicine, Karolinska Institutet, 141 86 Huddinge, Sweden; 7 Unit for Cell Signalling and SFI CAST, Oslo University Hospital, 0310 Oslo, Norway

**Keywords:** Tankyrase inhibitor, LGR5, WNT signalling, Intestine, Lineage tracing, Stem cell

## Abstract

**Background:**

The WNT pathway regulates intestinal stem cells and is frequently disrupted in intestinal adenomas. The pathway contains several potential biotargets for interference, including the poly-ADP ribosyltransferase enzymes tankyrase1 and 2. *LGR5* is a known WNT pathway target gene and marker of intestinal stem cells. The LGR5^+^ stem cells are located in the crypt base and capable of regenerating all intestinal epithelial cell lineages.

**Results:**

We treated *Lgr5*-*EGFP*-*Ires*-*CreERT2;R26R*-*Confetti* mice with the tankyrase inhibitor G007-LK for up to 3 weeks to assess the effect on duodenal stem cell homeostasis and on the integrity of intestinal epithelium. At the administered doses, G007-LK treatment inhibited WNT signalling in LGR5^+^ stem cells and reduced the number and distribution of cells traced from duodenal LGR5^+^ stem cells. However, the gross morphology of the duodenum remained unaltered and G007-LK-treated mice showed no signs of weight loss or any other visible morphological changes. The inhibitory effect on LGR5^+^ stem cell proliferation was reversible.

**Conclusion:**

We show that the tankyrase inhibitor G007-LK is well tolerated by the mice, although proliferation of the LGR5^+^ intestinal stem cells was inhibited. Our observations suggest the presence of a tankyrase inhibitor-resistant cell population in the duodenum, able to rescue tissue integrity in the presence of G007-LK-mediated inhibition of the WNT signalling dependent LGR5^+^ intestinal epithelial stem cells.

**Electronic supplementary material:**

The online version of this article (10.1186/s40659-017-0151-6) contains supplementary material, which is available to authorized users.

## Background

Regulation of the wingless-type MMTV integration site family member (WNT)/β-catenin pathway, is attenuated in several cancers [1]. Colorectal tumours, in particular, often show dysregulated WNT/β-catenin signalling. For example, mutations in the adenomatous polyposis coli (*APC*) tumour suppressor gene or the gene encoding β-catenin (*CTNNB1*) result in colon adenomas [2, 3] and hypermethylation of SFRP1, a WNT antagonist, occurs frequently in colorectal cancer [4]. Despite the critical role played by deregulated WNT/β-catenin signalling in several types of cancer, there are currently no direct WNT/β-catenin pathway inhibitors in clinical use. A major concern when targeting this pathway is the involvement of WNT/β-catenin signalling in stem cell proliferation, maintenance and fate decision in several tissues. Therefore, potential therapeutic effects of WNT/β-catenin pathway inhibitors have to be balanced with their possible impact on normal stem cell biology.

Various biotargets in the WNT/β-catenin signalling pathway have been used to develop pathway inhibitors (reviewed in [5]). In particular, porcupine inhibitors that regulate the release of WNT morphogens [6] and tankyrases have been explored [7–9]. In the presence of WNT ligands, the β-catenin destruction complex does not form, β-catenin escapes ubiquitin–proteasome degradation and translocates to the nucleus to enable transcription of WNT/β-catenin target genes. Tankyrase 1 and 2 are poly-ADP ribosyl transferases that modify target proteins by attaching ADP-ribose moieties that serve as recognition signals for poly-ubiquitination through the E3 ubiquitin ligase ring finger protein 146 (RNF146), [10, 11]. Tankyrase 1/2 are involved in numerous cellular processes including telomere elongation, mitotic progression, glucose metabolism and stress granule formation [12–14], in addition to positively regulating WNT signalling by promoting the degradation of AXIN1/2 [15]. Altered levels of tankyrase 1 and 2 have been reported in several types of cancers [16–20] and in astrocytoma, increased expression of tankyrse1 is associated with increased pathological grade [21]. Recently, several tankyrase inhibitors, including G-631, XAV939, JW55 and G007-LK have been identified [15, 22–25]. The specificity of G007-LK was validated using super-resolution and electron microscopy demonstrating that G007-LK promotes formation of membrane-free structures containing active components of the WNT/β-catenin destruction complex in colorectal cancer cell lines [9]. At the tankyrase catalytic domain, G007-LK binds the adenosine site and inhibits the growth of several colon cancer cell lines in vitro and in vivo [22–24, 26]. However, the effect of tankyrase inhibition on stem cell homeostasis in the crypt and on the integrity of the intestinal lining remains to be investigated. Any intestinal toxicity would be a potential contraindication for the systemic application of tankyrase inhibitors [24, 25].

WNT signalling is considered essential for tissue homeostasis in the intestine [27, 28] and intestinal morphology and function are maintained through the continuous renewal of epithelial cells. In the small intestine, the complete set of cells from the crypt base to the top of each villus, is regenerated in 5–7 days [29]. Lineage tracing experiments have shown that leucine rich repeat containing G protein coupled receptor 5 (LGR5) -expressing (LGR5^+^) crypt base columnar (CBC) cells are stem cells capable of regenerating all intestinal epithelial cell lineages [30, 31]. In mice, WNT-dependent proliferation of cycling CBC LGR5^+^ stem cells gives rise to short-lived transit-amplifying (TA) cells, which reside in the crypt for 2–3 days. The TA cells undergo several rounds of cell division when maturing into terminally differentiated cells upon reaching the crypt-villus border. Specific depletion of LGR5^+^ stem cells has shown that this cell population is not essential for maintaining the structure and function of the intestine [32]. After intestinal injury, label-retaining LGR5-negative cells might function as reserve stem cells in the crypts [32–35]. These reserve stem cells are referred to as quiescent intestinal stem cells (QISCs) and have been suggested to reside in the +4 position counting from the crypt base. Expression of BMI1 polycomb ring finger oncogene (BMI1) has been proposed as a marker for the +4 cell population [36], however, other intestinal cell populations also express BMI1, including LGR5^+^ CBC cells [37]. In addition, several other QISC population(s) markers have also been suggested, but their specificity is under debate [34, 35, 38].

Maintaining tissue homeostasis in the intestine is critical for several life functions. Here, we show that the herein tested orally administered doses of the tankyrase inhibitor G007-LK resulted in reduced proliferation of the LGR5^+^ stem cell population and reduced the number of lineage-traced offspring in the small intestine. Upon the administered doses, the inhibitor was well tolerated in mice, without disrupting the structure and function of the intestine.

## Methods

### Pharmacokinetic analysis

Medicilon Preclinical Research (Shanghai) LCC performed the pharmacokinetic studies; in brief, ICR mice (n = 25) from Sion-British SIPPR/BK Lab Animal Ltd, Shanghai were fed 100 mg G007-LK/kg chow enriched diet ad libitum for 3 consecutive days, body mass and food consumption was measured daily. Blood samples (approximately 500 µl) were collected via cardiac puncture after euthanasia by CO_2_ inhalation at 0 h (immediately after test diet removal), 1, 2, 4, and 12 h post dose (n = 5 mice pr. time point). Blood samples were placed in tubes containing sodium heparin and centrifuged at 6800×*g* for 6 min at 4 °C to separate plasma from the rest of the sample. The concentrations of G007-LK in plasma were determined using a high performance liquid chromatography/mass spectrometry/mass spectrometry (HPLC/MS/MS) method. Non-compartmental pharmacokinetic parameters were calculated using WinNonlin^®^ Professional 5.2 software.

### Animals, drug treatment and lineage tracing

**Fig. 1:**
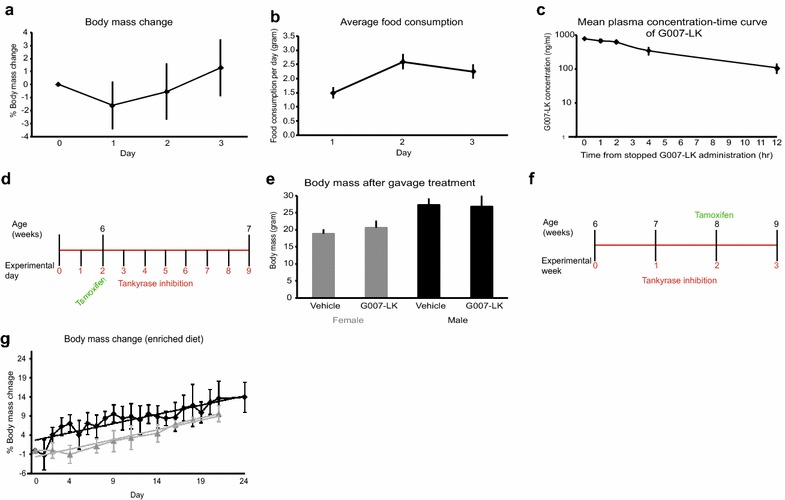
Body mass, administration schemes and pharmacokinetics of G007-LK. **a** Average relative change in body mass of ICR mice compared with body mass at starting day for 3 days treatment with G007-LK enriched diet (mean ± SEM, n = 25). **b** Average food consumption for ICR mice fed G007-LK enriched chow for 3 days (mean ± SEM, n = 25). **c** Mean plasma concentration time curve of G007-LK in mice following administration of G007-LK enriched chow (100 mg/kg chow) for 3 days (n = 5), measured at 0, 1, 2, 4 and 12 h after termination of G007-LK treatment (mean ± S.D). **d** Administration schema for G007-LK by oral gavage, short-term. Tamoxifen was administered by i.p. injection, when indicated, to induce lineage tracing from LGR5^+^ cells. **e** Body mass of mice measured at the end of the short-term G007-LK administration protocol (mean ± SEM, n ≥ 4). **f** Administration schema for G007-LK by enriched chow, long-term. Tamoxifen was administered by i.p. injection, when indicated, to induce lineage tracing from LGR5^+^ cells. **g** Average relative change in body mass of male mice compared to body mass at starting day for long-term treatment with G007-LK enriched diet (black line) and control treated mice (grey line) (mean ± SEM, n ≥ 3). Linear regression modelling of the body mass developments is indicated with straight lines, same colour coding as for the corresponding observed data

Drug treatment experiments were performed with wild type (wt) (FVB/N), single or double transgenic *Lgr5*-*EGFP*-*Ires*-*CreERT2;R26R*-*Confetti* mice [39], unless indicated otherwise. The tankyrase inhibitor, G007-LK, was administered orally either by gavage (10 or 50 mg/kg body mass once daily, vehicle: 15% dimethylsulfoxide [DMSO], 17.5% Cremophor EL, 8.75% Miglyol 810 N, 8.75% ethanol in phosphate buffered saline [PBS]) or in G007-LK enriched chow (100 or 1000 mg G007-LK/kg chow ad libitum, corresponding to a daily G007-LK dose of approximately 20 or 200 mg/kg body mass, respectively, for a mouse with a body mass of 25 g and consumption of approximately 5 g enriched diet/day), (Diet 5001, Research Diets, Inc.). G007-LK treatments were initiated at the age of 5 weeks and 5 days for oral gavage treatment or 6 weeks for enriched chow administration and continued for 9 or 21 days, respectively (Fig. 1d, f).

Lineage tracing experiments were conducted in male double transgenic (*Lgr5*-*EGFP*-*Ires*-*CreERT2;R26R*-*Confetti*) mice and initiated by activating inducible Cre-recombinase with one intraperitoneal (i.p.) injection of tamoxifen (0.225 mg tamoxifen/g body mass). For lineage tracing experiments, oral gavage administration of G007-LK was initiated 2 days prior to tamoxifen administration to ensure that G007-LK had a stable effect on the LGR5^+^ stem cells at the time of induced linage tracing. In separate lineage tracing experiments, administration of G007-LK enriched chow was initiated 2 weeks prior to activation of the Cre-recombinase by administration of tamoxifen. The body mass of the G007-LK-treated mice and the amount of consumed chow was measured 5 days per week. In both treatment regimes, G007-LK administration was maintained until the animals were sacrificed 7 days after tamoxifen administration. The mice were treated with G007-LK for 9 days by oral gavage or 21 days with the enriched diet. To determine the reversibility of the G007-LK-induced effects on the small intestine, a 2-day washout period of G007-LK was allowed prior to tamoxifen administration and activation of Cre-recombinase activity. The lineage tracing experiments in the absence of G007-LK were continued for 7 days until sacrifice.

All transgenic mice were bred and maintained within a specific pathogen free (SPF) barrier facility according to local and national regulations, and the Norwegian Animal Research Authority (FOTS ID #4385) approved all experiments in advance of their implementation.

### Confocal microscopy

Pieces of duodenum from mice that had undergone induced lineage tracing were fixed for 30 min in 4% paraformaldehyde (PFA) at room temperature, rinsed in PBS and immediately prepared for confocal microscopic analyses. Under a stereo microscope one row of intestinal villi was sectioned from the duodenal wall with a scalpel, rinsed in dH_2_O and embedded in Mowiol 4-88 (Sigma-Aldrich). The samples were examined with a Zeiss LSM 780 confocal microscope (Carl Zeiss MicroImaging GmbH) equipped with a multiline argon laser (458/488/514 nm), a 10 mW DPSS laser (561 nm), a 405-30 CW laser diode (405 nm), and a helium–neon (HeNe) laser (633 nm). The objective used was a Zeiss Plan-Apochromat 20×/0.8 DIC II. To calculate the numbers and volumes of the traced cells, confocal Z-stacks were analysed with Imaris 7.7.2 (Bitplane AG). A similar threshold for the segmentation of the traced cells was used in all Z-stacks examined. Image visualisations were performed with the basic Zeiss LSM 780 ZEN 2010 software, Photoshop CS4 (Adobe), and Imaris 7.7.2.

### Immunohistochemistry (IHC)

Mouse tissue was fixed overnight in 4% PFA, routinely processed and paraffin embedded. The paraffin-embedded tissue was sectioned at 3–4.5 μm. IHC staining was performed as previously described [40], with primary antibodies against β-catenin, green fluorescent protein (GFP), Lysozyme and Chromogranin A (rabbit monoclonal anti-β-catenin, Abcam ab32572; rabbit monoclonal anti-GFP, Cell Signaling Technology #2956; rabbit monoclonal anti-Lysozyme, Abcam ab108508; rabbit polyclonal anti-Chromogranin A, Abcam ab15160). For quantitative analyses of the IHC-stained tissue sections, the slides were scanned using the Hamamatsu Digital Slide Scanner NanoZoomer 2.0-HT C9600-13 and the number of positive and negative cells in up to 10 crypts or villi from each animal was counted manually. Periodic acid-Schiff (PAS) and Alcian blue staining was performed at the Department of Pathology, Oslo University Hospital, using standard protocols.

### Immunofluorescence labelling (IF)

Slides with tissue sections were deparaffinised in xylene and re-hydrated through a graded series of ethanol. Heat-antigen retrieval was performed in 10 mM sodium-citrate buffer supplemented with 0.05% Tween 20 in a pressure cooker for 3 min and the slides were slowly cooled down. Slides were rinsed in washing buffer (PBS supplemented with 0.1% Tween), incubated with blocking buffer (PBS supplemented with 5% goat serum and 5% bovine serum albumin (BSA)) for 30 min at room temperature and rinsed in washing buffer. Primary antibodies (mouse monoclonal anti-BrdU, ThermoFisher #B35128; monoclonal anti-GFP, Cell Signalling #2956) diluted in blocking buffer were applied and incubated over night at 4 °C. The slides were rinsed in washing buffer and secondary antibodies (goat anti-mouse secondary antibody, Alexa Fluor 488, ThermoFisher #A-11017; goat anti-rabbit secondary antibody, Alexa Fluor 594, ThermoFisher #A-11012) diluted in blocking buffer were applied for 2 h at 37 °C. The slides were rinsed in washing buffer and incubated with 4′,6-diamidino-2-phenylindole (DAPI, 1:1000 dilution in blocking buffer). The slides were mounted with Vectashield mounting medium with DAPI. Evaluation of the signals was performed in an epifluorescence microscope. Selected areas were photographed in a Zeiss Axioplan 2 microscope equipped with an Axio Cam MRM CCD camera and Axio Vision software.

### Bromodeoxyuridine (BrdU) labelling

Mice were treated with BrdU (100 mg/kg body mass) 1 h before being sacrificed or with daily 1 mg BrdU injections for 7 days prior to sacrifice. Duodenal tissue samples were fixed and processed as described above. BrdU-labelled cells were detected using a BrdU IHC kit (Abcam #125306) according to the manufacturer’s protocol, or using a monoclonal anti-BrdU antibody (ThermoFisher #B35128) and a Mouse-on-Mouse kit (Vector Laboratories #BMK-2202) according to the IHC protocol outlined above. The fraction of BrdU-labelled crypt cells was calculated.

### Gene expression array

Total RNA was isolated from fresh frozen duodenal tissue from control (vehicle gavage, n = 6; control chow, n = 3) and G007-LK treated (10 mg/kg gavage, n = 5; 50 mg/kg gavage, n = 2; 100 mg/kg chow, n = 3) mice. 40 ng total RNA was used in the Low Input Quick Amp Labeling protocol (Agilent Technologies) before hybridisation to the SurePrint G3 Mouse Gene Expression 8 × 60K Microarray (Agilent Technologies) according to the manufacturer’s protocol. Data were pre-processed and analysed using GeneSpring GX software, version 13.0 (Agilent Technologies). In brief, the mRNA probes were normalised to the 75 percentile and median-centred. The normalised data were filtered based on expression (20–100 percentile). Unsupervised hierarchical clustering was performed using the Euclidean algorithm for similarity measures and Ward’s rule for linkage.

### PCR and RT-qPCR

All experimental mice were ear marked and genomic DNA (gDNA) extracted from the ear punches was used for genotyping by qualitative PCR. The genotyping primer sequences were as follows: Lgr5-EGFP-Ires-CreERT2-FW (5′- CAC TGC ATT CTA GTT GTG G-3′) and human Lgr5-EGFP-Ires-CreERT2-Rev (5′- CGG TGC CCG CAG CGA G-3′); R26R-Confetti-FW (5′- GAA TTA ATT CCG GTA TAA CTT CG-3′) and R26R-Confetti-Rev (5′- AGA GTA TAA AAC TCG GGT GAG C -3′). The mRNA expression levels of genes of interest were determined by reverse transcriptase quantitative (RT-q)PCR using cDNA, constructed from mRNA isolated from duodenal tissue samples, as templates. TaqMan Assays (Primer/Probe sets) from ThermoFisher Scientific were used for gene specific RT-qPCRs; AXIN2 (Mm00443610_m1), LGR5 (Mm00438890_m1), Mki67 (Mm01278617_m1) and OLFM4 (Mm01320260_m1).

### Statistical analysis

Data are expressed as mean ± standard error of the mean (SEM) or standard deviation (S.D), as indicated. Unless stated otherwise, two-tailed Student’s *t* test was used, where p values ≤ 0.05 were considered significant.

## Results

### Pharmacokinetic properties of G007-LK and treatment of experimental mice

To address the pharmacokinetic properties of the tankyrase inhibitor G007-LK, ICR mice (n = 25) were treated for 3 days with a G007-LK-enriched diet, containing 100 mg G007-LK/kg chow. No obvious macroscopic abnormal phenotypes were observed and only minor, not statistically significant (*t* test, p > 0.05) changes in body mass of the treated animals were recorded during the treatment period (Fig. 1a). The food consumption was stable day 2 and 3 of the G007-LK enriched chow administration period, but statistically significant lower (*t* test, p < 0.05) the first day when the new chow was introduced (Fig. 1b). The consumed amount of enriched chow at day 2 and 3 resulted in a G007-LK daily dose of approximately 10 mg/kg body mass. Non-compartmental pharmacokinetic analysis showed that following three days of G007-LK-enriched chow administration, the mean C_max_ value (the first sampling point followed the mouse nocturnal eating phase, 0 h) was 762.5 ± 40.28 ng/ml (1.44 ± 0.08 μM) in plasma. Additional concentrations of G007-LK in plasma were measured 1, 2, 4 and 12 h after completion of G007-LK administration and the plasma concentrations at these time points were all statistically significant different from time point 0 h (Fig. 1c and Additional file 1: Table S1, *t* test, p < 0.01). The mean areas under the curve (AUC_(0-t)_) and AUC_(0-∞)_ were calculated to be 4119.80 and 4770.32 ng/ml*h, respectively and G007-LK t_1/2_ was 4.17 h. Using pharmacokinetic data from intravenous (IV) administration of G007-LK [26], the mean bioavailability (F) for G007-LK in the enriched chow was calculated to be 22.22%. Pharmacokinetic parameters of G007-LK following administration of enriched diet for three consecutive days are listed in Table 1.

**Table 1:** Pharmacokinetic parameters after administration of G007-LK enriched diet (n = 25 mice)

Diet containing G007-LK (100 mg/kg)						
Sex	t_1/2_ (h)	C_max_ (ng/mL)	AUC_(0-t)_ (ng/mL*h)	AUC_(0-∞)_ (ng/mL*h)	F^a^ (%)	F^b^ (%)
Female	4.17	762.50	4119.80	4770.32	22.22	20.69

t_1/2_: half life time, C_max_: maximum plasma concentration, AUC_(0–t)_: The integral of the concentration–time curve to time t, AUC_(0–∞)_: The integral of the concentration–time curve to time ∞, F: bioavailability

^a^The bioavailability was calculated as F (%) = (Dose_iv_ × AUC_po(0–t)_)/(Dose_po_ × AUC_iv(0–t_) × 100%

^b^The bioavailability was calculated as F (%) = (Dose_iv_ × AUC_po(0–**∞**)_)/(Dose_po_ × AUC_iv(0–**∞**_) × 100%

The tolerance of G007-LK treatment in the experimental mice used in this study was further evaluated by short-term oral gavage treatment daily for 9 days with 10 or 50 mg G007-LK/kg body mass (Fig. 1d). This G007-LK administration route did not statistically significant (*t* test, p > 0.05) affect body mass, measured at experimental endpoint, compared to vehicle treated animals (Fig. 1e). For long-term treatment, mice were fed G007-LK-enriched diets, 100 or 1000 mg G007-LK/kg chow for up to 3 weeks (Fig. 1f). Body mass development was not affected during the treatment period compared to control treated mice (Fig. 1g). Food consumption was constant (Additional file 2: Figure S1), resulting in daily doses of G007-LK up to 200 mg/kg body mass. Clinical observations revealed no abnormalities during the treatment period.

### G007-LK treatment repressed lineage tracing from LGR5^+^ intestinal stem cells

**Fig. 2:**
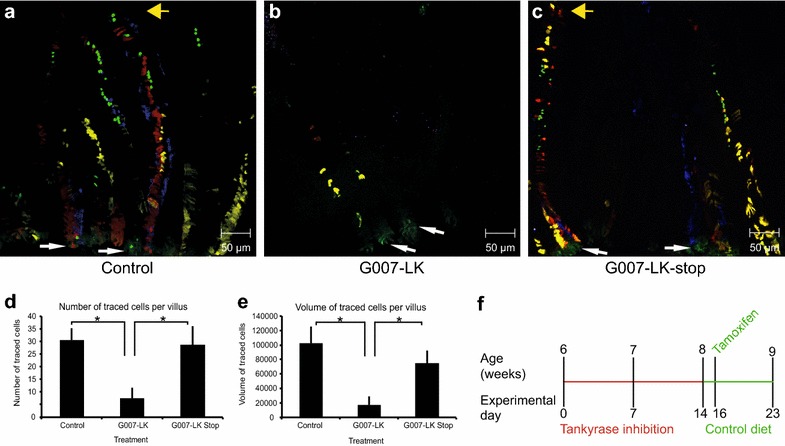
Lineage tracing from LGR5^+^ cells in the duodenum. **a**, **b** Confocal imaging of duodenal tissue in a *Lgr5*-*EGFP*-*Ires*-*CreERT2;R26R*-*Confetti* mouse after 7 days of lineage tracing in control (**a**) and G007-LK-treated, enriched chow 100 mg G007-LK/kg (**b**) mice according to the G007-LK administration schema outlined in Fig. 1f. LGR5^+^ stem cells express EGFP in the cytoplasm and are identifiable at the crypt base (white arrows indicate LGR5^+^ cells at the crypt base, yellow arrows indicate the tip of the villi). Traced LGR5^+^ cell progeny express RFP (red fluorescent protein), YFP (yellow fluorescent protein), EGFP (in the nucleus) or CFP (cyan fluorescent protein). **c** Confocal imaging of duodenal tissue in a *Lgr5*-*EGFP*-*Ires*-*CreERT2;R26R*-*Confetti* mouse after termination of G007-LK treatment (enriched chow, 100 mg G007-LK/kg chow, G007-LK-stop) prior to initiation of LGR5^+^ cell progeny tracing (administration schema outlined in **f**). **d** Quantification of the number of traced cells in Control, G007-LK and G007-LK-stop treated mice (mean ± SEM, *p < 0.05, n ≥ 3). **e** Quantification of the volume of traced cells in Control, G007-LK and G007-LK-stop treated mice (mean ± SEM, *p < 0.05, n ≥ 3). **f** Administration schema for G007-LK enriched chow in the G007-LK-stop group, G007-LK administration was stopped 2 days prior to initiation of lineage tracing by IP injection of tamoxifen

*Lgr5*-*EGFP*-*Ires*-*CreERT2;R26R*-*Confetti* double transgenic mice [39] were treated with vehicle (n = 8) or G007-LK (n = 5) and tamoxifen according to the two administration schemes outlined in Fig. 1d, f. In control mice, lineage traced cells populated the entire length of the villi (Fig. 2a) in the majority (69.9%) of the villi (Additional file 3: Figure S2A). Long-term treatment with G007-LK (100 mg/kg chow) resulted in markedly reduced lineage tracing with only a few cells derived from LGR5^+^ stem cells (Fig. 2b). Similar data were obtained after short-term treatment with G007-LK (10 mg/kg body mass), (Additional file 3: Figure S2B). Image analysis and quantification showed a significant (*t* test, p < 0.05) reduction of the number (Fig. 2d) and volume (Fig. 2e) of lineage traced cells after G007-LK treatment. The number of villi with traced cells was also significantly (*t* test, p < 0.05) reduced in G007-LK treated mice (Additional file 3: Figure S2A). The LGR5-expressing cells were easily identifiable by their cytoplasmic expression of GFP at the crypt base in control and G007-LK treated mice (Fig. 2a–c and Additional file 3: Figure S2C–H). Microscopic analyses of tissue sections stained with an antibody towards GFP (Additional file 3: Figure S2C–E) showed that the ratio of crypts with GFP positive cells versus the total number of counted crypts was reduced with 23% in the G007-LK treated compared with control mice (control mice n = 6, average ratio 0.51; G007-LK treated mice n = 10, average ratio 0.39, p = 0.05).

To determine whether the effect of G007-LK-mediated inhibition of tankyrases in LGR5^+^ cells was reversible, mice were fed G007-LK-enriched chow (100 mg G007-LK/kg chow) for 2 weeks. The diet was then reverted to a G007-LK-free diet 2 days prior to tamoxifen administration and induction of lineage tracing for 7 days until the mice (n = 3) were sacrificed (G007-LK-stop), (Fig. 2f). Confocal microscope analyses showed the presence of lineage traced cells throughout the entire length of the villi (Fig. 2c). Based on the confocal microscope images, the number and volume of the traced cells were calculated for each of the groups; Control, G007-LK and G007-LK-stop (Fig. 2d, e). One-way ANOVA tests showed that at least one of the groups (Control, G007-LK or G007-LK-stop) were significantly different (p < 0.05) from the other(s), both when the number and volume of traced cells were tested. Two sample *t* tests showed that both the control and G007-LK-stop groups were statistically significant different (p < 0.05) from the G007-LK group (Fig. 2d, e).

### G007-LK treatment specifically targets LGR5^+^ stem cells

**Fig. 3:**
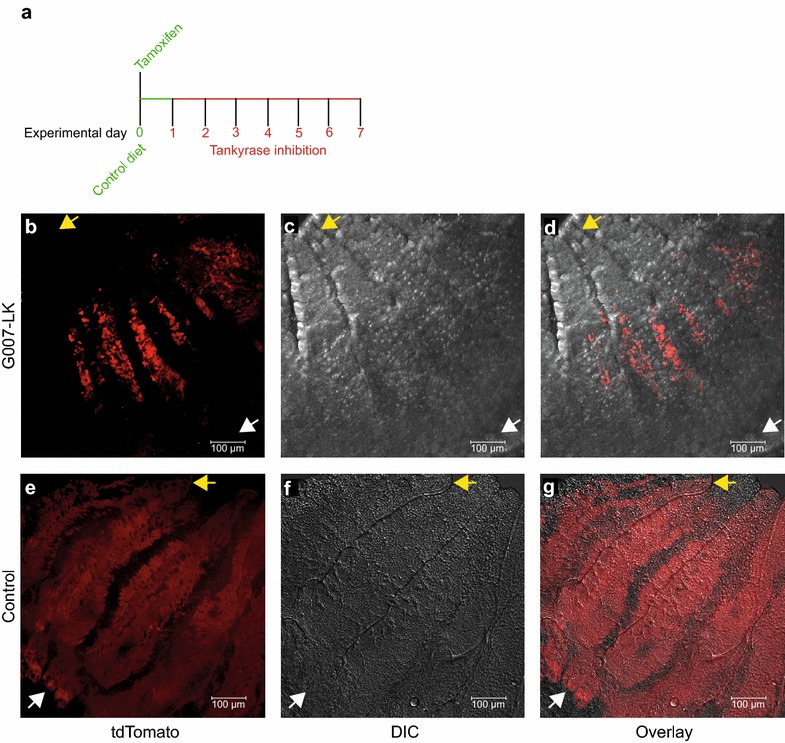
G007-LK specifically targets LGR5^+^ WNT-dependent intestinal stem cells. **a** Administration schema for G007-LK enriched chow 24-36 h after initiation of lineage tracing by IP administration of tamoxifen. **b** Confocal imaging of duodenal tissue in a *Lgr5*-*EGFP*-*Ires*-*CreERT2;R26R*-*tdTomato* mouse showing traced progeny expressing RFP (tdTomato) 7 days after initiation of lineage tracing, of which the last 6 days in the presence of G007-LK (enriched chow 100 mg G007-LK/kg). **c** Differential interference contrast (DIC) image of same section as in **b**. **d** Overlay of **b** and **c**. **e** Confocal imaging of duodenal tissue in a *Lgr5*-*EGFP*-*Ires*-*CreERT2;R26R*-*tdTomato* mouse showing traced progeny expressing RFP (tdTomato) 7 days after initiation of lineage tracing. The mice were fed control diet. **f** Differential interference contrast (DIC) image of same section as **e**. **g** Overlay of **e** and **f**. Yellow arrows indicate the tip of the villi and white arrows indicate the crypt bases

To further investigate the cellular specificity of G007-LK, administration of G007-LK was initiated 24–36 h after administration of tamoxifen to *Lgr5*-*EGFP*-*CreERT2;R26R*-*tdTomato* mice (Fig. 3a). After G007-LK administration (100 mg G007-LK/kg chow, n = 3 animals) for 6 consecutive days, lineage traced cells were detected in the middle of the duodenal villi (Fig. 3b–d). The lack of lineage traced cells in the crypts and lower regions of the villi suggests that the cells residing in this area of the duodenum originated from a non-recombined and tankyrase-independent stem/progenitor cell population. It is possible that the proliferation rate of these cells is lower than that of LGR5^+^ stem cells, since the lineage traced cells did not reach the villi tips, which was the case in control mice (Fig. 3e–g).

### Tankyrase inhibition reduced canonical WNT signalling and altered the proliferation rate of duodenal crypt base columnar cells

**Fig. 4:**
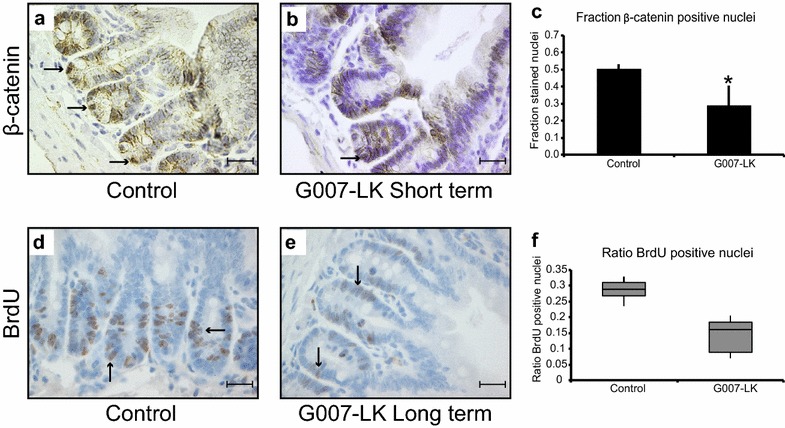
G007-LK treatment reduced canonical WNT signalling. **a**, **b** Immunohistochemistry (IHC) of duodenal tissue sections stained with an antibody against β-catenin, from control (**a**) and G007-LK-treated, gavage 10 mg G007-LK/kg body mass (**b**) mice (black arrows indicate non-Paneth cells in the crypt base with positive nuclear β-catenin staining). **c** Quantification of the fraction of β-catenin-positive nuclei among the crypt base cells in control and G007-LK-treated mice (mean ± SEM, *p < 0,05, n = 3). **d**, **e** BrdU incorporation, after 1 h incubation, into crypt base and transit amplifying (TA) cells in control (**d**) and G007-LK-treated, enriched chow 100 mg G007-LK/kg chow (**e**) mice (positive cells indicated with black arrows). **f** Median ratios of BrdU positive cell in the crypt of control and G007-LK treated mice (Box = 25th and 75th percentile, bars = min and max values) All images were taken with a 40× objective (scale bar 20 μm)

To verify that the administered doses of the tankyrase inhibitor had the expected effect on canonical WNT signalling, we stained duodenal tissue sections with a β-catenin-specific antibody. Nuclear localisation of β-catenin implied active canonical WNT signalling in vehicle (n = 3), (Fig. 4a) and G007-LK-treated (gavage 10 mg/kg, n = 2 and 50 mg/kg, n = 1) mice (Fig. 4b). The fraction of β-catenin positive nuclei in the crypt base cells was calculated and the results showed a significant (*t* test, p < 0.05) reduction in β-catenin-positive nuclei in G007-LK-treated compared to control mice (Fig. 4c).

Next, BrdU was administered to control and G007-LK-treated mice to determine whether G007-LK treatment influenced the proliferation of duodenal crypt cells. BrdU incorporation was observed in control and G007-LK treated mice (Fig. 4d, e). However, there was a significant (*t* test, p < 0.05) reduction in the fraction of BrdU-labelled crypt cells in G007-LK-treated mice compared to the control group (control, n = 4 animals, ratio of positive cells 0.28; G007-LK treatment, 100 mg/kg chow, n = 7 animals, ratio of positive cells 0.14; *t* test, p < 0.05), (Fig. 4f). Immunofluorescence labelling of duodenal tissue sections confirmed the higher frequency of BrdU labelled cells in the control mice compared to G007-LK treated mice (Additional file 4: Figure S3A and B). After repeated, long term (daily for 7 days) administration of BrdU (control n = 3; G007-LK 100 mg/kg chow n = 3), BrdU-labelled cells were observed at the tips of the villi in control mice (Additional file 4: Figure S3C) as well as in G007-LK-treated mice (Additional file 4: Figure S3D), indicating that certain stem cells are functional and dividing during treatment with G007-LK.

### G007-LK treatment did not alter duodenal morphology

**Fig. 5:**
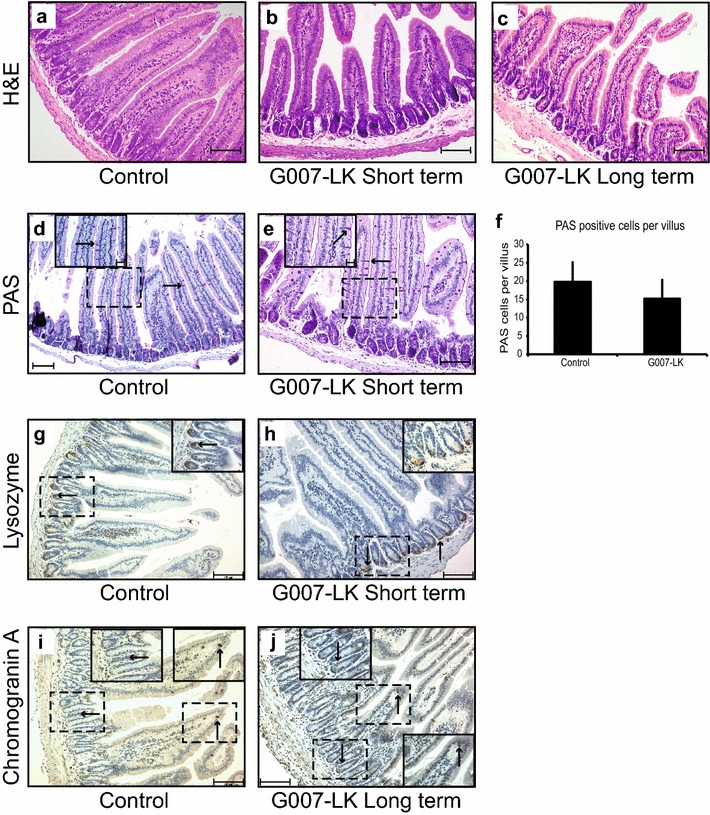
Morphology and cellular composition of duodenal tissue. (**a**–**c**) Haematoxylin and eosin (H&E)-stained sections from control (**a**), short-term gavage, 50 mg G007-LK/kg body mass (**b**), and long-term enriched chow, 100 mg G007-LK/kg chow (**c**) treated mice. **d**, **e** Periodic acid-Schiff (PAS)-stained cells (indicated with black arrows) were identified in duodenal tissue samples from control (**d**) and G007-LK (**e**) treated (gavage 50 mg G007-LK/kg body mass) mice (inset shows enlargements of boxed areas in main panel). **f** Quantification of the average number of PAS-positive cells per villus in duodenal tissue sections from control and G007-LK-treated mice (mean ± SEM, n = 3). **g**, **h** IHC of duodenal tissue sections, stained with an antibody against Lysozyme, from control (**g**) and G007-LK treated, gavage 10 mg G007-LK/kg body mass (**h**) mice (inset shows enlargements of boxed areas in main panel). (**i**, **j**) IHC of duodenal tissue sections, stained with an antibody against Chromogranin A (positive cells indicated with black arrows), from control (**i**) and G007-LK-treated, long term enriched chow, 100 mg G007-LK/kg chow (**j**) mice (inset shows enlargements of boxed areas in main panel)

Analyses of haematoxylin and eosin (H&E) stained formalin-fixed paraffin-embedded (FFPE) tissue sections showed that the gross morphology of the duodenum with respect to crypts and villi was maintained in G007-LK-treated mice (9 days oral gavage, 10 mg/kg, n = 17, 50 mg/kg, n = 2; 21 days enriched diet, 100 mg G007-LK/kg chow, n = 13, 1000 mg G007-LK/kg chow, n = 2) compared to control (vehicle, n = 7; control diet, n = 7), independent of G007-LK administration protocol (Fig. 5a–c). Detailed microscopic analyses of H&E stained proximal duodenal tissue sections from G007-LK gavage treated mice compared to control mice showed that there were no statistically significant (*t* test, p > 0.05) changes in crypt:villus ratios, crypt duplication rates or size, goblet cell number or size or crypts per villus base. Close examination of H&E stained crypts showed that G007-LK treatment did not alter the cellular composition of the crypt base, including the localisation of the Paneth and adjacent cells. PAS staining of glycoprotein containing goblet cells revealed that there was no significant difference (*t* test, p > 0.05) in the number of these cells between the vehicle and G007-LK treated mice (Fig. 5d, e, quantified in f). Similar data for the goblet cells were obtained by Alcian blue staining of acidic mucin (Additional file 5: Figure S4A–C). Detailed microscopic analyses of distal duodenum and ileum tissue sections showed that G007-LK treatment did not induce major morphological changes to the epithelial cell layer in these segments of the intestine. However, occasional crypt abscesses were observed in the G007-LK treated mice (Additional file 5: Figure S4D).

IHC staining of duodenal tissue sections with an antibody targeting Lysozyme confirmed the equal distribution pattern of Paneth cells in the crypt base in control and G007-LK treated animals (Fig. 5g, h). Analyses of tissue sections stained with an antibody towards Chromogranin A showed comparable distribution patterns of enteroendocrine cells in control and G007-LK treated mice (Fig. 5i, j).

The effect of G007-LK treatment on intact duodenal tissue was further addressed by gene expression analyses of control (n = 9) and G007-LK (n = 10) treated mice. Unsupervised hierarchical clustering analysis revealed two subgroups, which did not coincide with the treatment groups (Additional file 5: Figure S4E). No probes showed statistically significant differences (p > 0.05) between the vehicle and G007-LK-treated groups. This was also the case when a curated list of genes specific for the WNT signalling pathway was analysed for differential expression. Expression levels of specific genes were in addition addressed by qPCR (Additional file 5: Figure S4F) and confirmed that G007-LK treatment did not significantly (p > 0.05) influence global gene expression in duodenal tissue; implying equal cellular composition of the intestinal tissue in control and G007-LK treated mice. Any subtle, cell specific differences cannot, however, be excluded.

## Discussion

Deregulated WNT signalling contributes to the development and progression of many cancers, but very few WNT pathway antagonists have entered clinical trials (reviewed by Kahn [5]). This might relate to the lack of efficacy of WNT inhibitors in cancer cells per se, or to difficulties in the specific targeting of cancer cells without interfering with the vital roles of WNT signalling in maintaining tissue homeostasis and adult stem cells. In mice, small intestine tissue homeostasis is considered to depend on cycling, LGR5-expressing, WNT signalling-dependent cells located in the crypt base, that are capable of generating all intestinal epithelial cell lineages [30]. These cells also constitute the tumour initiating cells of intestinal cancers [41].

To clarify the effect of a tankyrase inhibitor, G007-LK, on intestinal tissue homeostasis, we have conducted in vivo lineage tracing experiments as well as histological analyses in transgenic mice. The tankyrase inhibitor was administered either by oral gavage or as part of an enriched diet. Here, we report that G007-LK is well tolerated by these mice (at daily doses up to approximately 200 mg/kg body mass) and that the LGR5^+^ intestinal stem cells are reversibly silenced in the presence of the inhibitor.

We observed no phenotypic changes in mice treated with G007-LK for up to 3 weeks. The treated mice had comparable body mass increase as control mice, food consumption was stable and as expected (approximately 5 g/mouse/day), and we observed no aberrant behavioural phenotypes. In adult mice, the WNT signalling pathway plays an important role in regulating several stem cell populations, including those in skin and intestine. The absence of phenotypic changes and the fact that there were no major effects on the gross morphology of the proximal duodenum in our experiments, suggest that in our models, the administered doses (approximately 20 or 200 mg G007-LK/kg mouse body mass) and administrative routes were well tolerated. In addition, 6 months treatment with G007-LK (158 mg/kg chow), although on a different, high fat-based diet, did not lead to visible toxic effects, but rather improved glucose tolerance and insulin sensitivity in parallel with increased plasma adiponectin levels [42]. Previous studies showed that intermediate and high doses (up to 100 mg G007-LK/kg body mass once daily for 14–20 days) of the tankyrase inhibitors G007-LK and G-631 administered either i.p. or orally, resulted in partially reversible focal necrosis and inflammation in the small intestine [24, 25]. In distal duodenum and ileum of the G007-LK treated mice we observed occasional crypt abscesses, which might be the first indication of a mild toxicity. However, we did not observe any other signs of toxicity such as villus blunting, ulceration or extensive inflammatory cell infiltration. As many cancers are dependent on WNT signalling, the inconsistency in reported results justifies further investigation of the toxicity and effects of tankyrase inhibitors, such as G007-LK, in preclinical and clinical studies.

The most pronounced effect of G007-LK administration observed in the current study was the markedly reduced lineage tracing from LGR5^+^ intestinal stem cells. Despite the inhibition of canonical WNT signalling, the gross morphology of the small intestine, including the crypts and villi, was not altered and the number, size and shape of the goblet cells were maintained. This might suggest a reduced turnover of functional intestinal cells, possibly triggered by delayed apoptosis of the differentiated cells at the tips of the villi. However, following extended treatment with BrdU (2 weeks of G007-LK treatment and 1 week with combined G007-LK and BrdU), BrdU-labelled cells were identified at the tips of the villi. This observation suggests the existence of one or more cell populations capable of generating new cells that differentiate into functional cells in the presence of a tankyrase inhibitor. A previous study demonstrated the dispensable role of CBC LGR5^+^ cells, since LGR5^+^ cell-specific depletion did not alter either the morphology or normal functioning of the intestine [32]. Several lines of evidence point towards the existence of additional stem/progenitor cell populations in the intestine, and it has been suggested that the +4 cells, located near the crypt base and characterised by BMI1, mTERT or HOPX expression, harbour stem cell properties [36, 43, 44], but the exclusiveness of the expression pattern of these markers and thus their usefulness as markers of intestinal stem cells are debated [32, 45–47]. Whether other facultative intestinal stem cells, (e.g., marked by BMI1, mTERT, LRIG1 or HOPX) are activated under conditions of tankyrase inhibition remains to be investigated.

Clinically effective inhibitors of the WNT signalling pathway could become important in future treatment of cancers with altered WNT signalling. A limitation of this study is the absence of in vivo experiments showing reduced tumour growth by the use of identical G007-LK administration routes. In the current study, we have shown that the tankyrase inhibitor, G007-LK, can be administered orally to mice without noticeable effects on duodenal morphology or function under the conditions tested. Furthermore, our data indicate that one or more tankyrase signalling-independent stem/progenitor cell populations are able to maintain overall duodenal tissue homeostasis, possibly at a reduced cellular turnover, in the absence of fully functional LGR5^+^ crypt base stem cells. Further research is required to identify these specific stem/progenitor cell population(s).

## Conclusion

Based on the herein presented in vivo lineage tracing data and morphological characterisation we conclude that doses of the tankyrase inhibitor G007-LK shown to be sufficient to inhibit tumour growth [24, 48] are well tolerated by mice within the time frames investigated. Lineage tracing from LGR5^+^ intestinal stem cells was reduced upon G007-LK treatment, without altering the main morphological characteristics of the intestine. Moreover, mice treated with G007-LK did not experience weight loss, suggesting that the absorptive capacity of the intestine was not negatively impacted. Our results suggest that G007-LK may be a candidate for use in preclinical trials to determine the efficacy of this drug in preventing growth of WNT dependent tumours.

## Additional files

**Additional file 1: Table S1.**Plasma concentrations of G007-LK in ICR mice (n = 5) following administration the G007-LK enriched diet for 3 consecutive days.

**Additional file 2: Figure S1.** Food consumption. (A) Average food consumption for mice fed G007-LK-enriched chow (100 mg G007-LK/kg chow) for 3 weeks. The grey curves represent average consumption per mouse for two animals housed in the same cage. The black curve illustrates the food consumption of one mouse housed alone.

**Additional file 3: Figure S2.** Percentage of villi with traced progeny and lineage tracing from LGR5^+^ cells. (A) Percentage of duodenal villi with traced progeny from LGR5^+^ cells in control, G007-LK and G007-LK-stop (enriched chow 100 mg G007-LK/kg chow) treated mice (mean ± SEM, * p < 0,05, n ≥ 3). (B) Confocal image of duodenal tissue in a *Lgr5*-*EGFP*-*Ires*-*CreERT2;R26R*-*Confetti* mouse after 7 days of lineage tracing in G007-LK-treated mice, gavage 10 mg G007-LK/kg body mass, G007-LK administration according to the schema outlined in Fig. 1d. LGR5^+^ stem cells express EGFP in the cytoplasm and are identifiable at the crypt base (white arrows). Traced LGR5^+^ cell progeny express RFP (red fluorescent protein), YFP (yellow fluorescent protein), EGFP (nuclear) or CFP (cyan fluorescent protein). The tissue section was counterstained with Hoechst dye for identification of the tissue morphology. (C-E) IHC staining with an antibody against GFP of duodenal tissue sections from control (C), G007-LK short-term, oral gavage, 10 mg G007-LK/kg body mass (D) and long-term, enriched chow, 100 mg G007-LK/kg chow (E) treated *Lgr5*-*EGFP*-*Ires*-*CreERT2;R26R*-*Confetti* mice, 7 days after initiating lineage tracing. All images in panels C–E were taken with a 4× objective (scale bar 200 μm). (F) 20× magnification of boxed area in C. (G) 20× magnification of boxed area in D. (H) 20× magnification of boxed area in E. All images in panels F–H were taken with a 20× objective (scale bar 50 μm)

**Additional file 4: Figure S3.** BrdU incorporation. (A and B) Immunofluorescence labelling of duodenal tissue sections from control (A) and G007-LK enriched chow, 100 mg G007-LK/kg chow (B) treated *Lgr5*-*EGFP*-*Ires*-*CreERT2;R26R*-*Confetti* mice, stained with DAPI (blue) and antibodies against BrdU (red) and GFP (green). BrdU and GFP double positive cells are indicated with yellow arrows, BrdU positive cells with pink arrows and GFP positive (LGR5 expressing) cells with white arrows. (C and D) IHC of duodenal tissue sections, stained for BrdU incorporation after daily treatments with BrdU for 7 days. Cells with incorporated BrdU (indicated with black arrows) were identified at the tips of the duodenal villi in control (C) and G007-LK (100 mg G007-LK/kg chow) treated (D) mice. Images are 20× representations from high resolution scanned tissues slides (scale bar 300 μm).

**Additional file 5: Figure S4.** Alcian blue-staining and gene expression analysis. (A and B) Alcian blue-stained cells were identified in control (A) and G007-LK-treated, oral gavage, 50 mg G007-LK/kg body mass (B) duodenal tissue samples (positive cells indicated with black arrows).
