# Supplementary material for: The tankyrase inhibitor G007-LK inhibits small intestine LGR5+ stem cell proliferation without altering tissue morphology
Source: Biol Res. 2018 Jan 9;51:3. doi: 10.1186/s40659-017-0151-6 (PMC5759193; doi:10.1186/s40659-017-0151-6)
Supplement: Supplementary file 1 — Additional file 1: Table S1.Plasma concentrations of G007-LK in ICR mice (n = 5) following administration the G007-LK enriched diet for 3 consecutive days. [file 40659_2017_151_MOESM1_ESM.docx]

Supplementary Table 1

Plasma concentrations of G007-LK in ICR mice (n=5) following administration the G007-LK enriched diet for 3 consecutive days

| **Time (h)** | **Diet Contain G007-LK** | | | | | | | | |
| --- | --- | --- | --- | --- | --- | --- | --- | --- | --- |
|  | **G007-LK Concentration in plasma (ng/mL)** | | | | | | | | |
|  | **G1-1** | **G1-2** | **G1-3** | **G1-4** | **G1-5** | **Mean** | **S.D.** | **t-test (p-value)** |  |
| **0** | 773,96 | 795,28 | 756,04 | 696,04 | 791,2 | **762,5** | **40,28** |  |  |
| **1** | 676,41 | 696,8 | 608,34 | 669,24 | 689,94 | **668,15** | **35,15** | 0,004254205 |  |
| **2** | 639,46 | 649,54 | 610,19 | 589,18 | 567,65 | **611,2** | **34,1** | 0,0002067 |  |
| **4** | 335,27 | 378,3 | 216,65 | 474,86 | 316,33 | **344,28** | **94,02** | 1,65028E-05 |  |
| **12** | 52,75 | 105,7 | 112,65 | 158,02 | 111,12 | **108,05** | **37,38** | 4,25302E-09 |  |
